# Editorial: Towards the wellbeing economy: economic, social, and environmental impact on mental wellness

**DOI:** 10.3389/fpsyt.2023.1228355

**Published:** 2023-06-13

**Authors:** Doreen W. H. Au, Yi Sun, Ho Ting Wong

**Affiliations:** ^1^School of Nursing and Health Studies, Hong Kong Metropolitan University, Hong Kong, China; ^2^Department of Building and Real Estate, The Hong Kong Polytechnic University, Hong Kong, China; ^3^Department of Business Administration, College of Management, National Taiwan Normal University, Taipei, Taiwan

**Keywords:** wellbeing economy, economic, environmental, social, mental wellness

A “wellbeing economy” is a new economic model that prioritizes environmental and human wellbeing over material and financial aspirations, with the goal of achieving sustainability by ensuring long-term wellbeing for all. Policymakers, businesses, and civil society are all becoming more involved in this concept. Several national governments have used the “wellbeing economy” as a guiding framework for creating development policies and measuring social and economic advancements in recent years. While the “wellbeing economy” shares some fundamental concepts with various post-growth conceptualizations, its language and concepts tend to be more adaptable to different social and economic contexts, permeating policy processes, and connecting to a variety of cultural traits, not just in advanced but also in developing economies (1).

By considering the “wellbeing economy” as a new way to assess economic and social value, the 193 member nations of the UN General Assembly developed the global action plan that later took the form of the Sustainable Development Goals (SDGs) for the years 2015–2030 (2). The SDGs laid out a grid of significant societal issues, each linked to a set of concise and quantifiable economic, social, and environmental targets. In going beyond material growth, the “wellbeing economy” recognizes, protects and promotes the contributions of natural, social, and human capital to collective wellbeing. The gross domestic product, which merely adds the market value of material production and consumption, is no longer an appropriate indicator of development for a “wellbeing economy”; instead, it calls for an interdisciplinary approach to measuring the state of natural ecosystems, looking into the underlying causes and connections of the problems, as well as their dynamic effects on physical and mental wellness.

This Research Topic includes two research articles and two review articles that discuss a variety of issues critical to our understanding of the components required to achieve the “wellbeing economy”. The aim is to gather ideas and evidence for an economic system that provides everyone with equal opportunities for advancement, as well as possible sustainable paths toward improved both physical and mental wellness. Each chapter reflects diverse scientific viewpoints from disciplines to provide a comprehensive glance on the issues.

In terms of environmental challenges, Thoma et al. described the root causes and interconnections of the climate and environmental crisis on mental health from a clinical psychology perspective. They outlined the potential underlying emotional, cognitive, and behavioral pathways, as well as how the climate and environmental crisis can amplify an individual's biopsychosocial vulnerability. Their review paper raises awareness of the growing psychopathology caused by anxiety, frustration, and despair as a result of the ongoing climate and environmental crisis, which includes climate change, pollution, environmental degradation, and/or destruction of the air, soil, water, and ecosystems.

Two research articles focus on the social challenges and examined the interconnections of external stimuli such as the life event crisis or perceived social isolation or loneliness on mental health (Zhang et al.; Chau et al.). Zhang et al. pointed out that although negative life event are stressful for individuals, the quality of life of individuals who experience life event crisis does not necessarily decrease. How individuals respond to the life event crisis is influenced by many factors and their study found that individual social capital system involving emotional and financial support could act as a buffer against the loss of an individual's wellbeing. They believe that social capital, as a kind of external social resource, could improve individual's emotional experience with life event crisis by taking positive coping styles to face the negative influence of life events. From their study, the establishment of a good interpersonal network are encouraged.

Along with the similar line of research, Chau et al. considered loneliness as a multidimensional phenomenon involving intimate, relational and collective dimensions and identified the putative psychological mechanisms that may contribute to the co-occurrence of loneliness dimensions with psychopathologies development including depression, social anxiety, and paranoia. They concluded that the core schemas (i.e., beliefs about the self and others) contribute to the heterogeneous patterns of expression of loneliness and mental symptoms.

In terms of economic challenges, Micai et al. raised the issue of equity issue and focus on people with intellectual disability and/or people with mental health conditions. Their review provided an overview of the personal budget, which can be given directly to the beneficiary or managed by an intermediary from social or public healthcare, who is delegated to purchase services based on the individual's needs. The personal budget approach can promote autonomy and empowerment by enabling people with intellectual disabilities and/or mental health conditions to actively participate in their care. Beneficiaries might also have more flexibility and control over their healthcare providers.

We anticipate that the articles in this Research Topic will contribute to our growing understanding of not only the various aspects of the “wellbeing economy”, but also of the intricate relationships that exist between them and how they affect the realization of the meta-purpose of “sustainability”, namely improved long-term wellbeing for all, enabling us to suggest a coherent sense of what the most urgent actions should be.

## Author contributions

DA prepared the initial draft. All authors listed have made a substantial, direct, and intellectual contribution to the revision and approved it for publication.
